# Supplementary material for: Clay-Catalyzed Ozonation of Organic Pollutants in Water and Toxicity on Lemna minor: Effects of Molecular Structure and Interactions
Source: Molecules. 2022 Dec 27;28(1):222. doi: 10.3390/molecules28010222 (PMC9822386; doi:10.3390/molecules28010222)
Supplement: Supplementary file 1 [file molecules-28-00222-s001.zip › molecules-1986565-supplementary.pdf]

Clay-catalyzed ozonation of organic pollutants in water and toxicity on *Lemna Minor*- Effects of molecular structure and interactions

Eric Noel Foka Wembe, Amina Benghafour, David Dewez and Abdelkrim Azzouz

SUPPORTING INFORMATION

1. Adsorption solutions and reaction mixture analysis

Table S1. HPLC-UV operating conditions of the Agilent-Cary 60 device (Agilent Technologies, USA)

	DAZ	DCF	BPA
Injection (µL)	2.0	0.2	2.0
Methanol (% vol.)	65	60	70
Eau (% vol.)	35	40	30
Mobile phase throughput (mL/min)	0.18	0.15	0é15

2. Clay behavior in aqueous media

Table S2. Some features of organic substrate in aqueous solutions and corresponding clay suspensions

Substrate	pK_a	pHs^a	Clay mineral added	pH_i^b	pH_f^c	Particle size (nm)	Zeta potential (mV)	Adsorption yield (%)[d]
BPA	9.6	5.49	Fe(II)Mt	3	4.31	1859	-23.5	0
				6	4.53	1366	-38.14	58
				9	5.33	1336	-37.31	0
				12	12.96	3128	-70.00	0
			HMt-1	3	4.25	727	-19.64	54
				6	4.18	1468	-29.42	62
				9	4.82	2039	-11.78	11
				12	12.90	1009	-11.54	0
DAZ	2.6	5.48	Fe(II)Mt	3	3.83	697	-41.52	10
				6	4.89	1485	-29.06	89
				9	6.01	1699	-41.56	47
				12	13.08	2332	-44.00	6
			HMt-1	3	4.28	1662	-9.83	14
				6	4.73	1495	-49.83	26
				9	5.30	858	-51.71	24
				12	13.14	852	-42.74	0
DCF	4.2	4.78	Fe(II)Mt	3	1.76	826	-70.91	85
				6	3.30	1692.1	-23.49	94
				9	4.58	2041.5	-51.96	79
				12	10.67	822.1	-50.46	0
			HMt-1	3	1.89	862.5	-86.43	93
				6	3.22	812.4	-28.17	95
				9	4.52	1018.8	-30.56	87
				12	10.64	963.3	-54.24	0

[a] pHs is the intrinsic pH of the solution of organic species before catalyst addition.
[b] pH_i is the initial pH of the solution of organic species after addition of catalyst and after adjustment by acid or base addition.
[b] pH_f is the final pH of the clay suspension in an organic species solution after 10 minutes of contact time.
[d] Adsorption yield (((Co-Ct)/Co)x100%) measured after a 30 min contact time.

3. Adsorption kinetics on clay surface

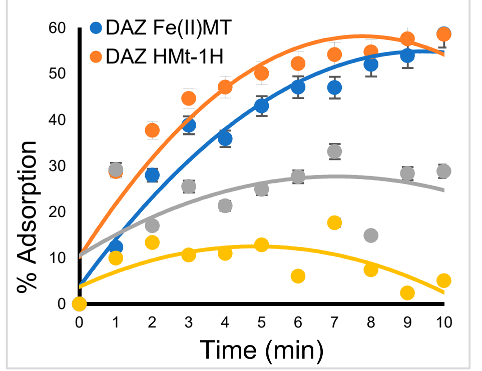

a. Diazinon at 250 nm

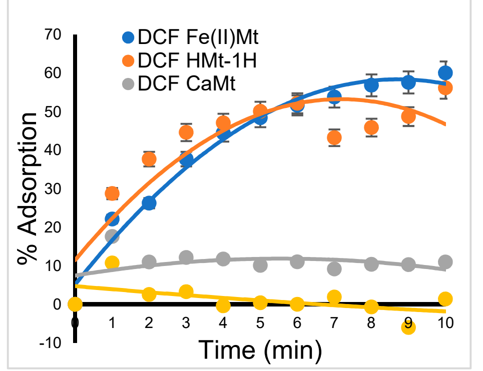

b. Diclofenac sodium at 275 nm

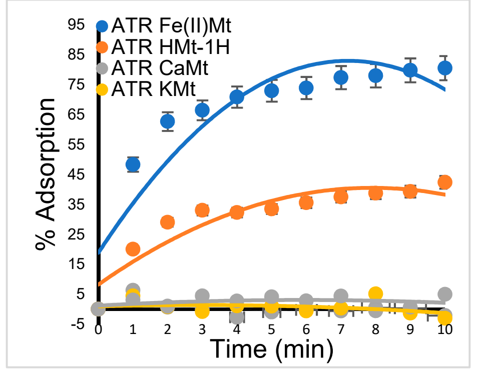

c. Atrazine at 220 nm

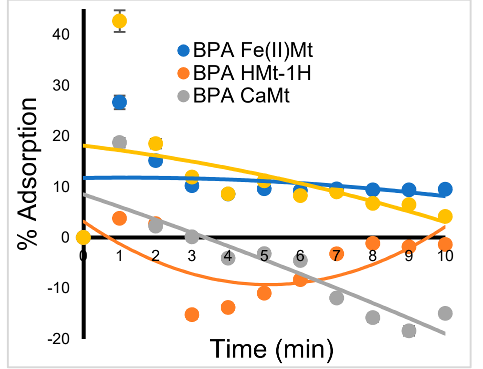

d. Bisphenol A at 275 nm

Figure S1. Evolution in time of the adsorption rate on different clay catalysts. The adsorption rate was expressed in terms of UV-Vis absorbance after 10 min contact time at intrinsic pH of the aqueous solution. Catalyst amount = 40 mg; $V_{Aliquot}$ = 30 mL. Adsorption rate (%) = 100× $\frac{(A_0-A)}{A_0}$. A_o and A are the initial and instant absorbances of the centrifuged supernatant.

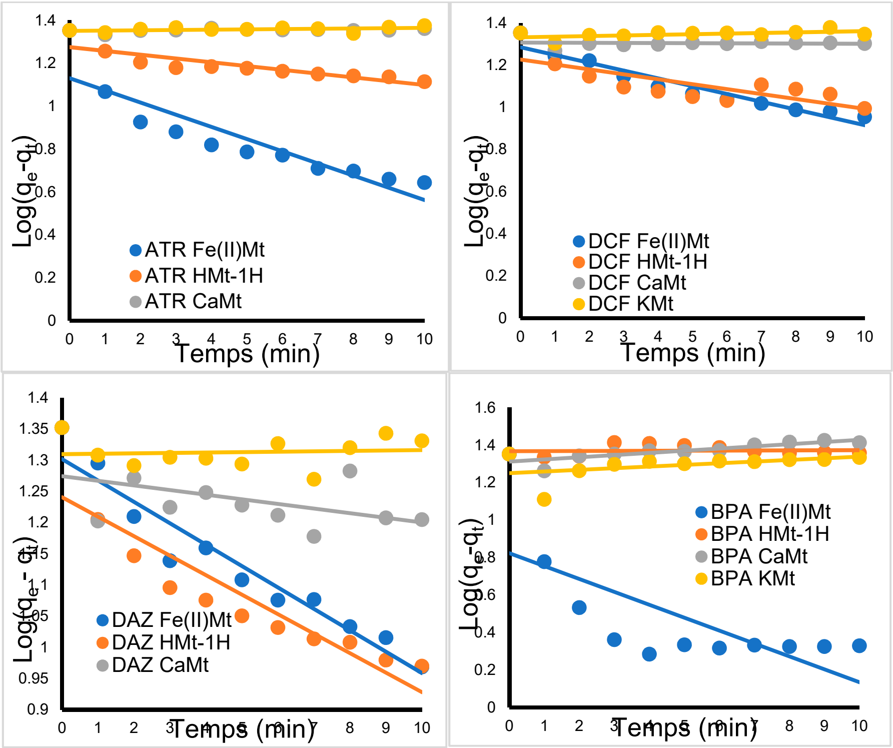

Figure S2. Pseudo-first order plot for organic substrate adsorption on different clay minerals. Adsorption was achieved at intrinsic pH of aqueous clay suspension; Volume = 30 mL; Initial concentration of organic substrate = 30 mg L^{-1}; T = 21± 2°C; adsorbent amount = 40 mg; Contact time = 10 min.

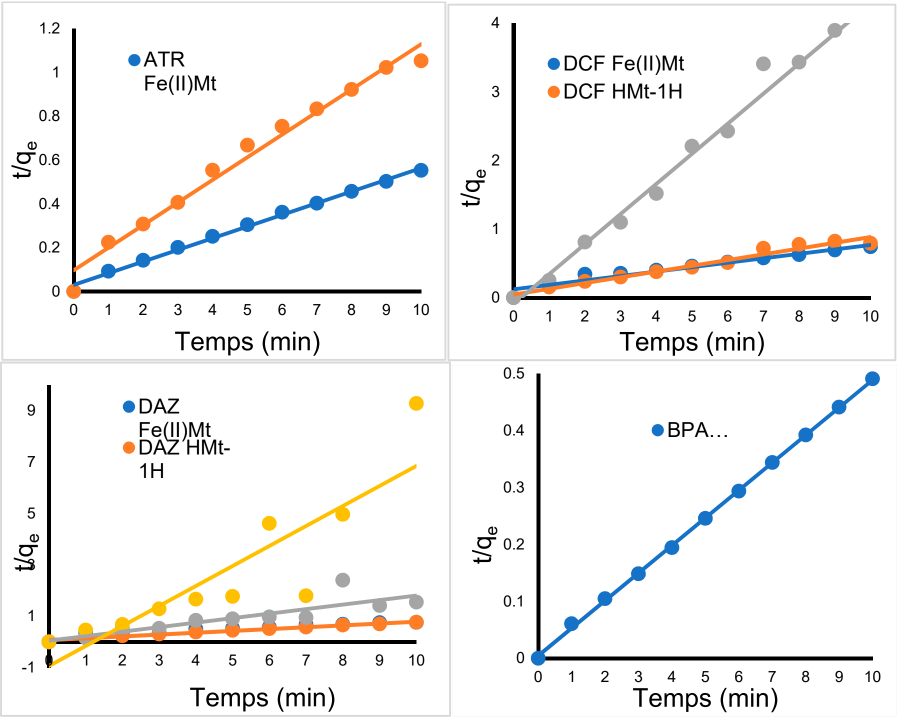

Figure S3. Pseudo-first order plot for organic substrate adsorption on different clay minerals. Adsorption was achieved at intrinsic pH of aqueous clay suspension; Volume = 30 mL; Initial concentration of organic substrate = 30 mg L^{-1}; T = 21± 2°C; adsorbent amount = 40 mg; Contact time = 10 min.

4. Adsorption equilibrium

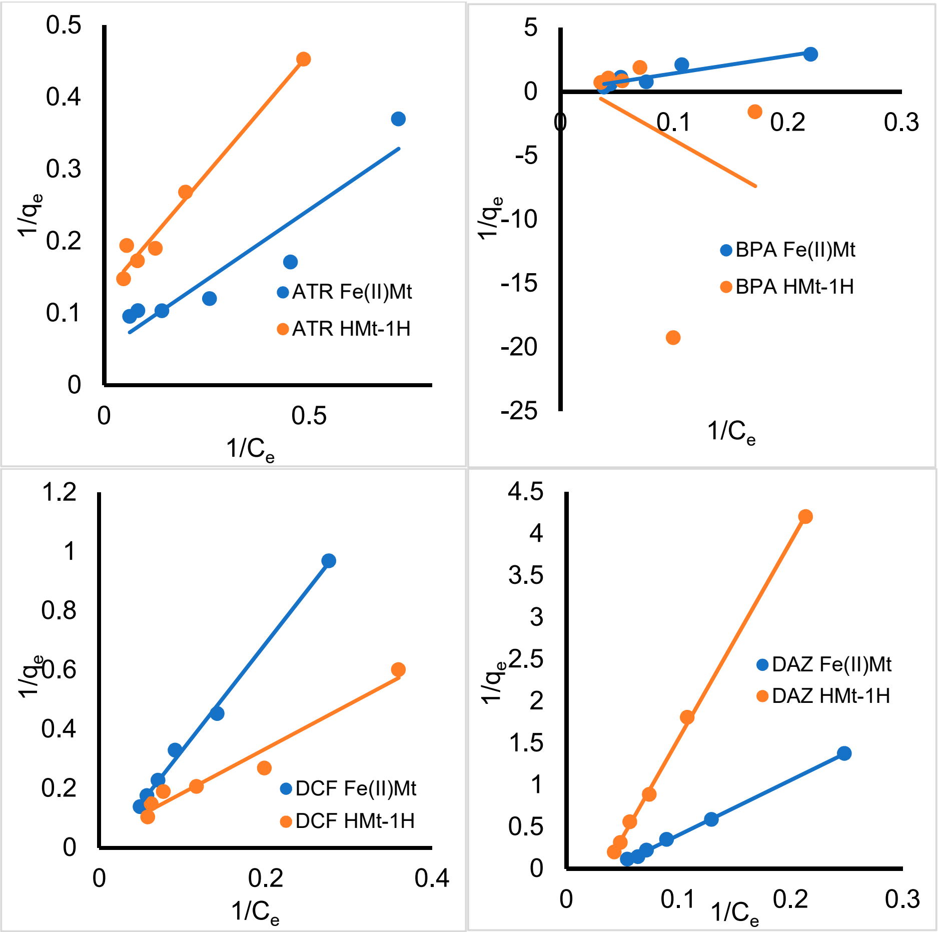

Figure S4. Langmuir's model plot for organic substrate adsorption on different clay minerals. Adsorption was achieved at intrinsic pH of aqueous clay suspension; Volume = 30 mL; Initial concentration of organic substrate = 30 mg L^{-1}; T = 21± 2°C; adsorbent amount = 40 mg; Contact time = 10 min.

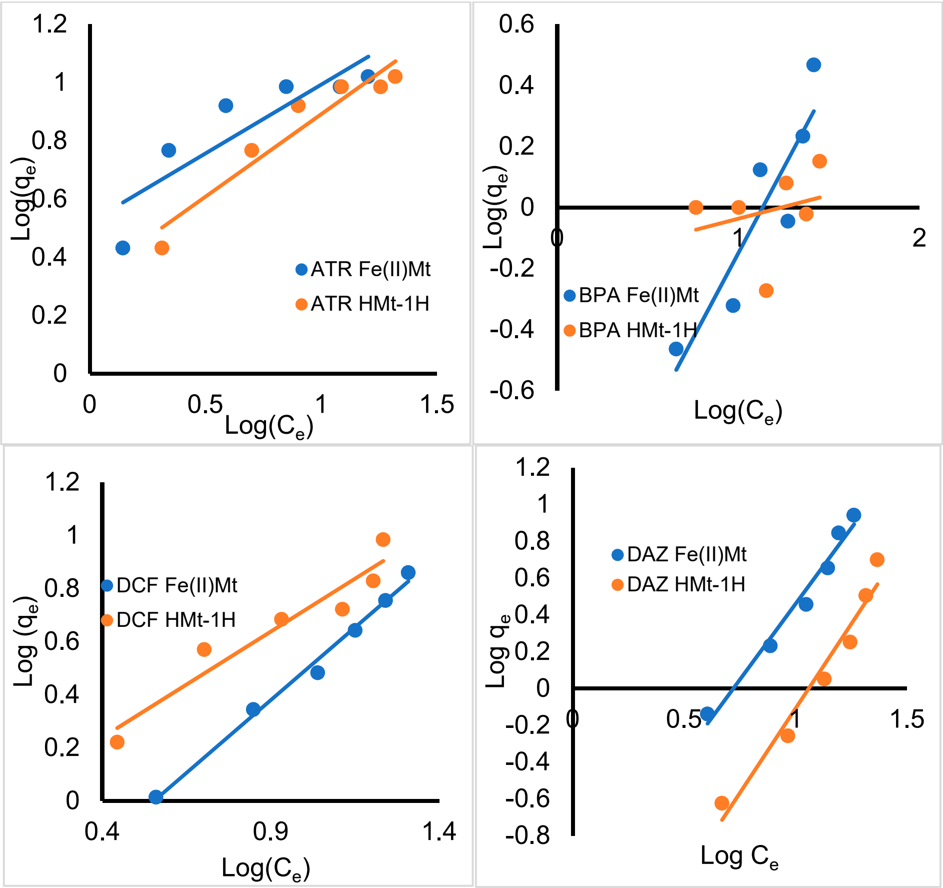

Figure S5. Freundlich's model plot for organic substrate adsorption on different clay minerals. Adsorption was achieved at intrinsic pH of aqueous clay suspension; Volume = 30 mL; Initial concentration of organic substrate = 30 mg L^{-1}; T = 21± 2°C; adsorbent amount = 40 mg; Contact time = 10 min. The highest values of the correlation coefficient were obtained when applying Langmuir's.

5. Toxicity of ozonized reaction mixtures

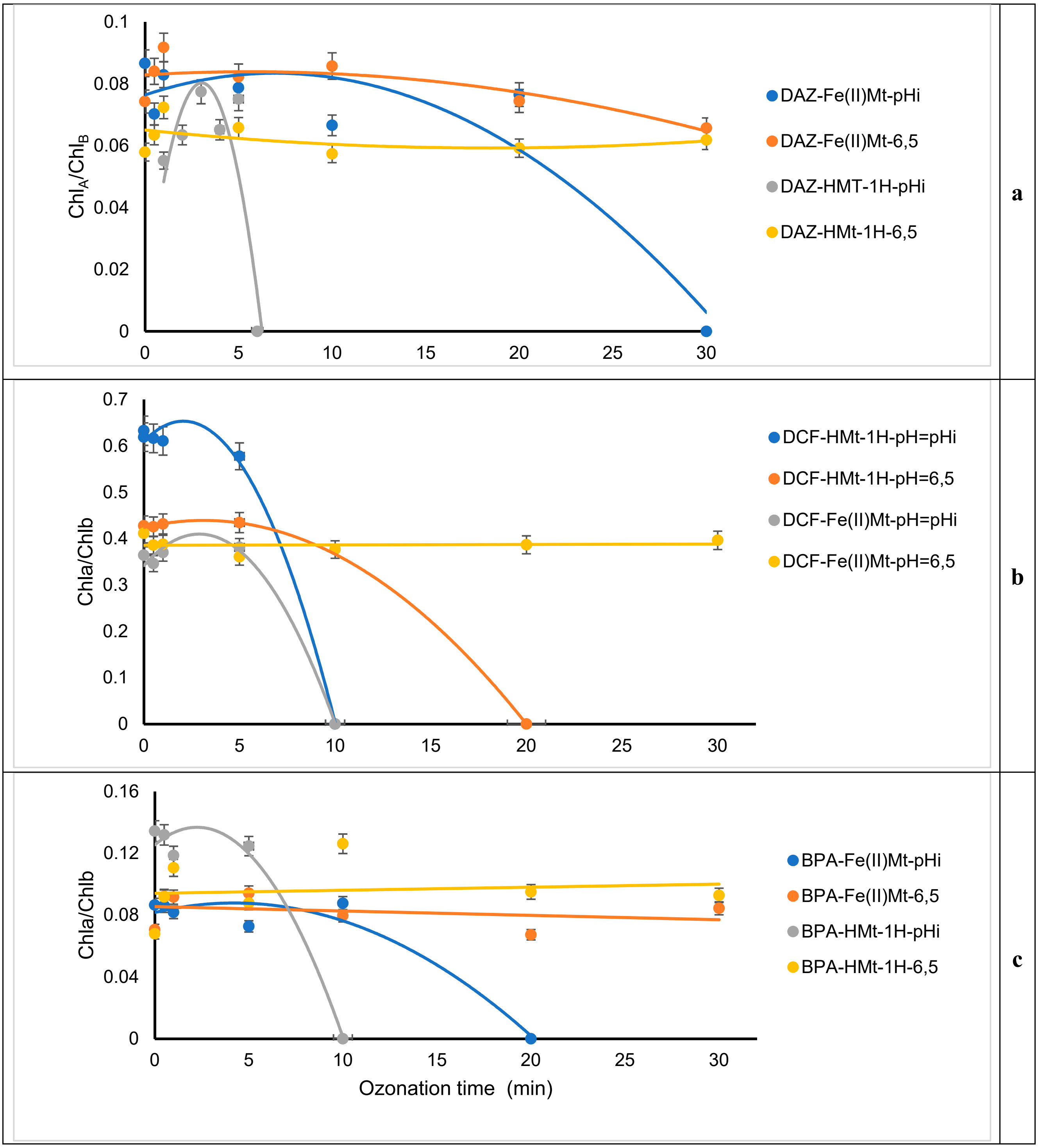

Figure S6. Effect of ozonation time on chlorophyll A-to-chlorophyll B ratio. The Chl_A/Chl_B ratio was measured by spectrophotometry at 620 and 665 nm based on Lichtenthaler-Buschman equations. The toxicity tests were achieved ***i.*** at constant pH 6.5 adjusted after ozonation and ***ii.*** at pH_i, the pH of the SIS mixture with the solution of the organic substrate before and after ozonation at different times.
